# Cluster Size Statistic and Cluster Mass Statistic: Two Novel Methods for Identifying Changes in Functional Connectivity Between Groups or Conditions

**DOI:** 10.1371/journal.pone.0098697

**Published:** 2014-06-06

**Authors:** Alex Ing, Christian Schwarzbauer

**Affiliations:** Aberdeen Biomedical Imaging Centre, University of Aberdeen, Aberdeen, Scotland, United Kingdom; Universiteit Gent, Belgium

## Abstract

Functional connectivity has become an increasingly important area of research in recent years. At a typical spatial resolution, approximately 300 million connections link each voxel in the brain with every other. This pattern of connectivity is known as the functional connectome. Connectivity is often compared between experimental groups and conditions. Standard methods used to control the type 1 error rate are likely to be insensitive when comparisons are carried out across the whole connectome, due to the huge number of statistical tests involved. To address this problem, two new cluster based methods – the cluster size statistic (CSS) and cluster mass statistic (CMS) – are introduced to control the family wise error rate across all connectivity values. These methods operate within a statistical framework similar to the cluster based methods used in conventional task based fMRI. Both methods are data driven, permutation based and require minimal statistical assumptions. Here, the performance of each procedure is evaluated in a receiver operator characteristic (ROC) analysis, utilising a simulated dataset. The relative sensitivity of each method is also tested on real data: BOLD (blood oxygen level dependent) fMRI scans were carried out on twelve subjects under normal conditions and during the hypercapnic state (induced through the inhalation of 6% CO_2_ in 21% O_2_ and 73%N_2_). Both CSS and CMS detected significant changes in connectivity between normal and hypercapnic states. A family wise error correction carried out at the individual connection level exhibited no significant changes in connectivity.

## Introduction

Functional connectivity MRI has become a widely used method for investigating human brain networks in health and disease; its potential in cognitive neuroscience and clinical research has been demonstrated in a large number of neuroimaging studies [1], [2].

Investigating the functional connectivity between all grey matter voxels makes full use of the connectional information available in the data. However, this approach results in a very large number of connectivity values, as illustrated by the following example: The total grey matter volume of the brain is approximately 675 ml [3]. Carrying out an fMRI scan at a typical spatial resolution of 3×3×3 mm results in approximately N = 25,000 grey matter voxels. Mapping the connectivity between all voxels gives rise to an N×N matrix of connectivity values. For a undirectional measure of functional connectivity, such as the widely used Pearson product-moment correlation coefficient, the connectivity matrix is symmetric and the number of unique elements is given by N(N-1)/2. In the present example, this corresponds to approximately 300 million connections. Functional connectivity is typically compared between different experimental conditions or groups of subjects. While the computational demands associated with a statistical comparison across all connectivity values are largely met by current high performance computer systems, there is a statistical challenge associated with the number of tests carried out. In the present example of 300 million unique connections, the application of an uncorrected probability threshold of p = 0.001 would lead to 300,000 false positives. Standard methods used to control the false positive rate (Type I error), such as the false detection rate (FDR) or the family wise error rate (FWER), perform well in the context of conventional task-related fMRI [4]. However, these methods are likely to result in insufficient statistical power when applied to such a large number of multiple comparisons [5].

A simple solution to address the multiple comparison problem is to reduce the number of tests that are carried out. This can be achieved by parcellating the cortex into anatomical regions of interest (ROI) [6], termed nodes. Comparing connectivity between cortical regions rather than individual voxels reduces the total number of comparisons. However, even when correcting over a smaller number of tests, standard type 1 error controlling procedures such as Bonferroni and false discovery rate (FDR) have been shown to be lacking in statistical power in this context [5].

In most functional connectivity studies, the multiple comparison problem is tackled by comparing univariate ‘connectivity maps’ consisting of N voxels, rather than connectivity matrices consisting of N×N elements. This approach is formally equivalent to the comparison of univariate parametric maps in task-based fMRI. Consequently, standard methods used to control the false positive rate (e.g., FDR or FWER) can be applied [4].

Univariate connectivity maps can be produced in a number of different ways. Seed-based connectivity mapping is one of the most widely used methods [7]. Here, functional connectivity is calculated between a reference voxel or region – also known as a ‘seed’– and every other voxel in the brain. This results in a univariate map, which is characterised by a single value per voxel. A limitation of this approach is that changes between groups or conditions can only be identified in relation to the seed. The selection of a specific seed may bias the result.

Independent component analysis (ICA) [8], [9] is another method that is frequently used to produce univariate connectivity maps. The advantage of ICA compared to the seed-based approach is that it is data driven. However, changes in connectivity can only be detected in relation to particular ICA components selected by the researcher; the identification of ICA components that match across conditions can be challenging.

In recent years, graph theoretical measures of functional connectivity have become increasingly popular [1], [10]–[12]. Graph theoretical summary measures [13] can be used to generate voxel-wise representations of whole brain connectivity. For example, the weighted global connectivity of a voxel is defined as the mean of that voxel's connectivity with every other voxel under analysis. In analogy to the seed-based approach or ICA, weighted global connectivity can be mapped across the whole brain to produce univariate connectivity maps. These maps can then be compared between different groups or conditions. A general limitation of this approach is the inherent reduction in information; a single value is used to quantify the functional connectivity between a voxel and the rest of the brain [14]. The resulting loss in information is a limitation to many comparative analyses.

**Figure 1:**
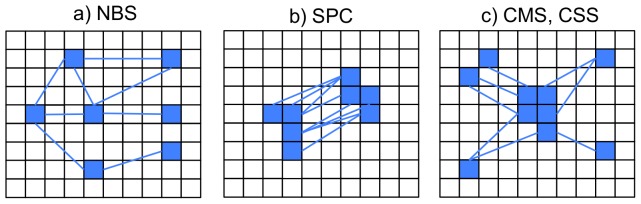
Connectivity clustering methods. Typical patterns of connectivity change that the different clustering methods are sensitive to. Here, squares represent voxels and lines represent thresholded connectivity changes. (a) NBS cluster size is defined by the number of thresholded connectivity changes forming continuously connected components. The NBS cluster above has an extent of eight. (b) SPC cluster size is defined by the number of thresholded connectivity changes forming pairwise spatial clusters. The SPC cluster above has an extent of eight. (c) CMS size is defined by the total number of connectivity changes between a spatially distinct cluster, and the rest of the brain. The CMS cluster above has an extent of eight. CSS size is defined by the spatial extent of a cluster, where each voxel in the cluster exhibits at least one connectivity change with another voxel in the brain. The CSS cluster above has an extent of five.

Recently proposed methods, such as the network based statistic (NBS) [5] or spatial pairwise clustering (SPC) [15], rely on a conceptually different approach to the multiple comparison problem. In these methods, ‘clusters’ of connectivity changes are considered in the statistical comparison of connectivity matrices. These procedures utilise the same statistical framework as conventional clustering methods in task-based fMRI [16]–[18]. Both methods involve an initial comparison between connectivity matrices, giving a matrix of test statistics. However, instead of assessing the significance of each test statistic, a cluster forming threshold is applied across the matrix. If the connectivity change between two brain areas exceeds this threshold, these regions form a ‘cluster link’. Connectional clusters are defined from these links. In contrast to task-based clustering procedures, which always measure activation in some way, connectivity clustering methods give very different information, depending on how clusters are defined. NBS and SPC provide very different information on connectivity change. This is similar to the way in which different graph theoretical measures can quantify diverse aspects of network topology [1]. In NBS, a cluster is defined as a set of brain regions continuously connected by pairwise cluster links (see fig 1a). In SPC, start and end points of links are grouped into clusters using a spatial vicinity criterion (see fig 1b); using this definition, connectivity can be considered to be altered between two specific brain regions. Similar to conventional clustering methods in task-based fMRI, permutation testing is used to obtain the statistical significance of clusters at a family wise error corrected level [17]. NBS and SPC have been successfully applied in a number of recent studies [19]–[23]. In cases where the contrast of interest leads to the formation of connected network structures, NBS has been demonstrated to provide an increase in statistical power compared to connection-wise family wise error control [5].

A limitation of NBS is that connectivity changes are not localisable to a particular region of the brain. Only the network as a whole, rather than its individual components, can be considered significant. Furthermore, the application of NBS on a voxel-by-voxel level is suboptimal; NBS does not explicitly model the spatial smoothness that is intrinsic to BOLD fMRI data. SPC does utilise this smoothness. However, SPC requires a cluster search over an enormous N(N-1)/2×N(N-1)/2 matrix [24]. In the case of our previous example, consisting of N = 25,000 voxels, a cluster search over a matrix consisting of 10^17^ elements would be required. The application of SPC on a voxel-by-voxel level is therefore challenging from a computational point of view.

In the present investigation, we introduce two new cluster based statistics to control for comparisons made over all connectivity values. These statistics are termed the Cluster Size Statistic (CSS) and the Cluster Mass Statistic (CMS). CSS is defined as the voxel-wise extent of a spatially continuous cluster, where each voxel in the cluster possesses at least one cluster-link with other voxels in the brain. CMS is defined as the total number of cluster-links between a spatially continuous cluster, and the rest of the brain (see fig 1c). Both CSS and CMS are able to identify changes in connectivity between a single region of the brain, and all other voxels under analysis. These methods are closely related to analyses involving the comparison of seed based connectivity maps. CSS and CMS can be regarded as an extension of seed based mapping to the whole brain. Any significant results obtained from studies utilising CSS/CMS methods can be used to guide the hypotheses of future seed-based investigations.

CSS and CMS measures have the advantage over NBS in that connectivity changes can be localised to a particular region of the brain. They also make full use of the spatial correlation that is intrinsic to fMRI data. They have the advantage over SPC that a cluster search over an N(N-1)/2×N(N-1)/2 matrix is not required. However, these analyses also provide information on connectivity change that is fundamentally different from what is offered by SPC or NBS. CSS and CMS statistics are designed to identify areas of the brain that show significant global connectivity change between groups or conditions. Clusters that are identified by CSS/CMS will not be detected by SPC or NBS, and vice versa.

Following a description of the principles underlying these tests, we demonstrate the performance of these methods in a simulation. The relative sensitivity of CSS and CMS methods is then tested on a real dataset by comparing BOLD fMRI scans carried out under normocapnic and hypercapnic conditions. The hypercapnic state is known to alter BOLD based measures of functional connectivity [25], [26] and therefore provides a convenient tool for testing the proposed CSS and CMS methods in the context of a repeated measures design.

## Theoretical Background

### Conventional cluster size thresholding in fMRI

Cluster size thresholding was introduced to fMRI by Poline et al [27], whose ideas were built upon by Forman et al [18]. This kind of test was initially applied to fMRI activation studies using Monte Carlo simulations. It was later adapted for use within a permutation based framework [17]. The basic idea underlying this kind of test is to use cluster size as the statistic of interest, in place of a voxel-wise test statistic. In a permutation based framework, any suitable statistic can be used to test for significance [28].

In a task-based fMRI study, the test proceeds in the following manner: For each permutation of the experimental labels, an image of voxel-wise test statistics (e.g., t-statistics) quantifying group activation is generated. This image is thresholded at a user-defined, cluster-forming threshold. The size of the largest cluster of voxels above this threshold is recorded. Voxels are considered to be a part of the same cluster if they are connected by a ‘face’ or an ‘edge’ (this is known as 18-connectivity). This procedure is repeated for many permutations of the experimental labelling. In this way, a maximal cluster size distribution is constructed from the data [17]. The experimental data is thresholded at the same t-value as the permutation data. Any clusters above the 100(1-p)th percentile of the maximum cluster size distribution are considered statistically significant.

### Cluster size statistic (CSS) and cluster mass statistic (CMS)

In this section, two novel cluster based statistics are described. These methods can be regarded as an extension of conventional cluster size testing. The following description is made with reference to a comparison between experimental conditions (repeated measures design); this is the experimental design used in the present investigation. However, this theoretical framework can also be applied to a comparison between two or more groups (cross-sectional design).

The first stage of both CSS and CMS methods is to map the functional connectivity between all voxel pairs in the brains' grey matter. Connectivity is mapped for all subjects, under both conditions. T-statistics are calculated between connectivity values taken under different experimental conditions. However, instead of using the Student t-distribution to assess the significance of connectivity changes, a cluster-forming threshold is applied across all test statistics. This is similar to the way in which a cluster-forming threshold is applied in conventional activation studies. T-values exceeding the initial threshold are termed ‘cluster links’, CSS and CMS statistics are defined from these cluster links. CSS is defined as the size of a spatially distinct cluster, where each voxel in the cluster possesses at least one cluster-link with other grey matter voxels. CMS is defined as the total number of cluster-links between voxels in a distinct cluster, and the rest of the brain (see fig 1c).

**Figure 2:**
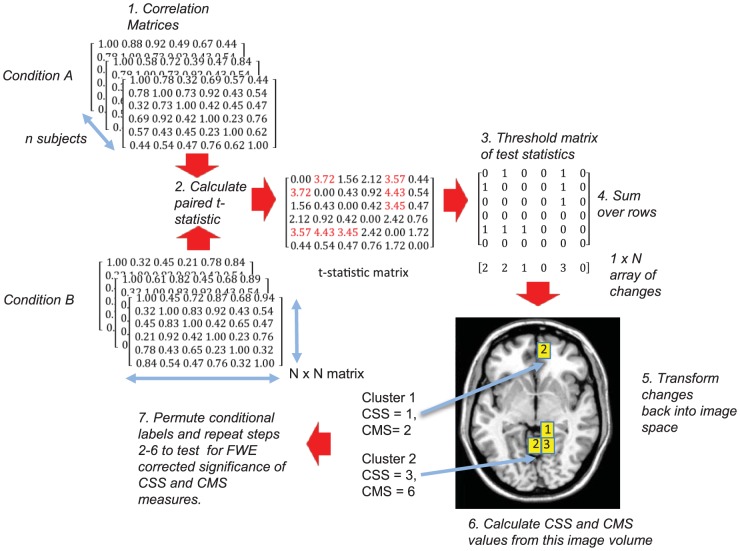
CMS/CSS methods. Illustration of data processing workflow for CSS and CMS measures. Note: The matrices are limited in size and only contain positive values for ease of exposition.

The workflow these procedures follow is illustrated in fig. 2 and described in detail below: 

**1**. Connectivity matrices are calculated for each subject for both experimental conditions A and B. **2**. An initial test statistic (e.g., t-statistic, f-statistic) is calculated for each matrix element to give a matrix of test statistics. **3**. In analogy to conventional cluster size thresholding methods used in fMRI, a cluster-forming threshold is applied to this matrix to create a binary matrix (termed an adjacency matrix in graph theoretical parlance). Both positive and negative cluster-forming thresholds are required when both increases and decreases in connectivity are of interest. In this case, steps 4-6 are carried out for each of two binary matrices produced using positive and negative cluster forming thresholds. Each column/row of the matrix is associated with a particular voxel, the values in the column/row denote the connectivity of that voxel with all other voxels under analysis. **4**. The sum is taken over each row of the binary matrix to produce an array of length N, where N denotes the number of voxels under investigation. Values in the array denote the number of connectivity changes a voxel has with the rest of the brain, which exceed the cluster-forming threshold. **5**. This array is transformed back into image space. This results in a univariate map where each voxel value denotes the number of connections with other voxels in the brain that exceed the cluster-forming threshold (cluster links). A voxel is considered to be part of a cluster if it exhibits at least one cluster link with another voxel in the brain. Clusters of connected voxels are defined on the basis of an 18-connectivity criterion [29] this means that they must share a face or an edge in image space (18-connectivity). **6**. In CSS, the cluster extent, characterised by the number of voxels forming the cluster, acts as the statistic of interest in determining a significant effect. In CMS, voxel intensity values comprising spatially distinct clusters are summed to give the total number of connectivity changes between a cluster, and the rest of the brain. **7**. Permutation testing [28] is used to identify clusters that are statistically significant at a probability corrected for family-wise errors. For each permutation of the experimental labels, the largest cluster in the associated image space is recorded. In this manner, a distribution of maximum cluster sizes is generated. Maximum cluster size distributions for CSS and CMS statistics are both generated in this way. For a one-sided test, any CMS or CSS clusters in the experimental data with cluster size values above the 100(1-p)th percentile of their respective maximum distributions, are considered to be statistically significant at a probability p [28]. When a two-sided test is carried out, clusters exceeding the 100(1-p/2)th percentile of the permutation distribution are considered statistically significant at a probability p. The maximum cluster-size distribution is the same for positive and negative cluster forming thresholds.

### Pseudo-thresholding the correlation matrix

A general problem with cluster size testing (this includes NBS and SPC) is the arbitrary nature of the cluster-forming threshold [4], [18]. Although results are valid for any initial threshold, its choice can have a real effect on the sensitivity and spatial specificity of the approach. Using a lower cluster-forming threshold favours the identification of larger, spatially extended clusters. Conversely, a high cluster-forming threshold supports the identification of spatially restricted, focal changes. In conventional, task based activation studies, threshold choice can be informed by the results obtained from previous studies. As this is a novel procedure, that is not possible here. In the present investigation, we take a different approach to threshold selection.

Here, we take two extreme cases to illustrate how the sensitivity and specificity of a cluster-based procedure can be affected by initial threshold choice. In permutation-based cluster tests, an initial threshold is set, and the permutation distribution is used to obtain a critical cluster size that defines significance in the experimental labelling. If the cluster-forming threshold is set very low, the critical cluster size could encompass most of the brain; this will severely limit the spatial specificity of the method. Conversely, setting the initial threshold very high could result in a critical cluster size smaller than the smoothing in the data. In the most severe case, the critical cluster size could be the size of one voxel/connection. In this situation, the ‘borrowing power’ of neighbouring voxels is not utilised. This is likely to limit the statistical power of the method: statistically, this would be very similar to a single voxel/connection test. Neither of the cases described in the text above is desirable.

**Figure 3:**
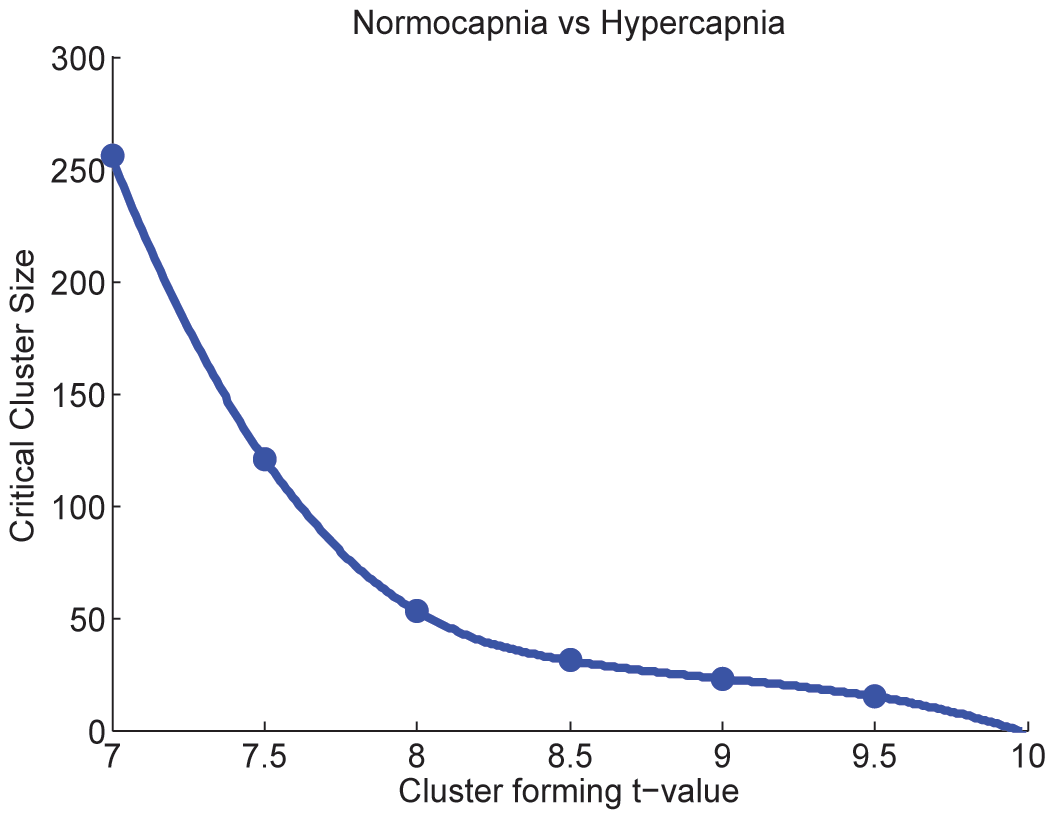
Plot of initial t-threshold against critical cluster extent. Plot of initial t-threshold (p<0.05 FWE corrected) against critical cluster extent. A cubic spline curve was fitted to the calculated data points.

Carrying out multiple tests to decide on an initial threshold post hoc is not viable as it is associated with an increased risk of false positives. Here, we describe a simple procedure to help guide the initial threshold choice. Using this method, we obtain the critical cluster size (CSS extent) associated with a particular cluster-forming threshold, without ‘looking’ to determine whether there are any significant clusters in the experimental labelling. Critical cluster size is then plotted against cluster forming threshold in an isocontour significance plot (this is similar to Friston et al [30] where these plots were derived mathematically for use in activation studies). In this way, it is possible to obtain information on the cluster size associated with an initial threshold before the data is tested. This procedure is not a solution to all of the problems associated with cluster size thresholding. Rather, the method offers a framework that can be used to obtain an initial threshold, which avoids the two extremes given as examples above. This is especially useful in situations where no previous studies exist to guide the initial threshold choice. This procedure is described in detail below:

1. Multiple permutation distributions are calculated from the data for a range of initial threshold values. Each of these distributions produces a critical cluster size. The critical cluster size is the p<0.05 FWE corrected significance level associated with a particular cluster-forming threshold. Clusters in the data that are larger than this critical size can be declared significant at a probability corrected for family wise errors. In order to reduce computational complexity, a smaller subset of the permutation distribution can be used in place of the full set [17].
2. Each critical cluster size is then plotted against the cluster-forming threshold associated with it; an example based on the data from the present study is shown in fig. 3. The plot is fitted with a spline curve.
3. This plot provides information on the cluster threshold and extent combinations that are required for statistical significance in the experimental labelling.

The curve in fig. 3 represents a constant (p>0.05, FWE corrected) probability of significance as the cluster-forming threshold is varied. Isocontour plots can be constructed for any other significance level by plotting the initial threshold against the appropriate critical cluster size.

The choice of exactly what cluster-forming threshold to use is still somewhat arbitrary. However, we argue that this procedure allows the experimenter to make a more informed choice regarding initial threshold selection. Using this procedure provides the experimenter with information on what *combination* of threshold and extent are required for clusters in the experimental labelling to reach statistical significance. This information is similar to that provided by Forman et al [18] for activation studies: Here, statistically significant combinations of initial threshold and cluster size were tabulated for other researchers to use (these tables implicitly assume identical cluster distributions between studies). This knowledge allows the threshold choice to be guided by hypothesis to a greater extent: For example (with reference to fig. 3), if focal effects are expected, or a high degree of spatial specificity is required, a cluster threshold above nine should be used. Conversely, if more diffuse clusters are expected, a lower initial threshold should be applied. At the same time, it is possible to avoid the two extreme cases given in the text above by avoiding thresholds that are associated with clusters that take up a large part of the brain, or are smaller than the smoothness in the data. The issue of ‘double dipping’ [31] does not arise, as this procedure does not give any information on the significance of clusters in the experimental labelling.

This method utilises the spatial correlation that is intrinsic to the data to guide the selection of the cluster-forming threshold. It is therefore reasonable to use this threshold selection procedure for both the CSS and CMS methods. However, it should be noted that this cluster selection procedure is not an integral feature of CSS or CMS and any initial threshold can be used with these methods.

### Simulation

Receiver operator characteristic (ROC) plots were constructed from simulated data to test the efficacy of CSS and CMS methods. The performance of these procedures was evaluated in the detection of a known ‘ground truth’ contrast, corrupted by noise. ROC curves are constructed by plotting the true positive rate (TPR) against the false positive rate (FPR) of contrast detection, across a range of discrimination thresholds. The discrimination threshold determines whether a test is classified as ‘true’ or ‘false’. In the present investigation, the discrimination threshold is defined by CSS/CMS cluster size.

Standard ROC methodology is designed to deal with single inferences (e.g. is a contrast present at a single voxel? yes/no). In this framework, TPR and FPR both have straightforward definitions: TPR is the proportion of true positives identified from all actual positives; FPR is the proportion of false positives out of all actual negatives. When multiple tests are carried out simultaneously, a number of different TPR and FPR definitions exist: free response receiver operator characteristic (FROC) [32] curves are constructed by plotting the proportion of true and false positive rates across all tests together. The alternative FROC (AFROC) [33] uses a different FPR definition: the probability of a false positive *anywhere* in the image. As CMS and CSS methods aim to control the family-wise error rate (the chance of one or more false positives anywhere in the image), the more stringent AFROC procedure is more meaningful in the current context.

The true positive rate was calculated by comparing a pure noise dataset with a dataset containing a known contrast. Calculating the false positive rate from this comparison is problematic when the test has a spatial aspect [34]: CSS and CMS control the family-wise error rate at the cluster level. As spatial smoothing is applied to the simulated data, the contrast is likely to blur into neighbouring voxels/connections. This creates a problem in determining what is ‘true’ background, which makes FPR calculation problematic. Following the procedure proposed by Smith and Nichols [34], the false positive rate was calculated here by comparing two noise only datasets. Calculating the FPR from this comparison provides exactly what is required in standard null hypothesis testing: the probability of a false positive in the presence of no real contrast.

The simulation presented here was carried out using a single brain slice. A 2-D slice was used in place of the whole brain to account for the computational demands posed by CSS and CMS statistics: Using the full 3-D dataset, each individual simulation run took over an hour on an Linux machine with 48 GB of memory and eight cores, each with 2.56 GHz of processing power, this time was decreased dramatically by reducing the simulation to two dimensions (∼2 minutes per run). A necessarily restricted 8-connectivity scheme was used to define spatially distinct clusters. All other aspects of the analysis remain the same using two in place of three dimensions. As in Zalesky et al 2010 [5], the contrast formed a pattern of connectivity change that the cluster methods were specifically designed to identify (i.e. a change in connectivity between a spatially restricted cluster, and the rest of the brain). Note that it does not make sense to compare CSS/CMS methods to the connection-wise test in this evaluation, as the single connection test measures something fundamentally different from what is quantified by CSS/CMS. The sensitivity of CSS and CMS are compared to that of the single connection test in comparisons made on real data, as this does not require the specification of a contrast.

The pre-processing carried out on the simulated data was the same as that used on the experimental data.

**Figure 4:**
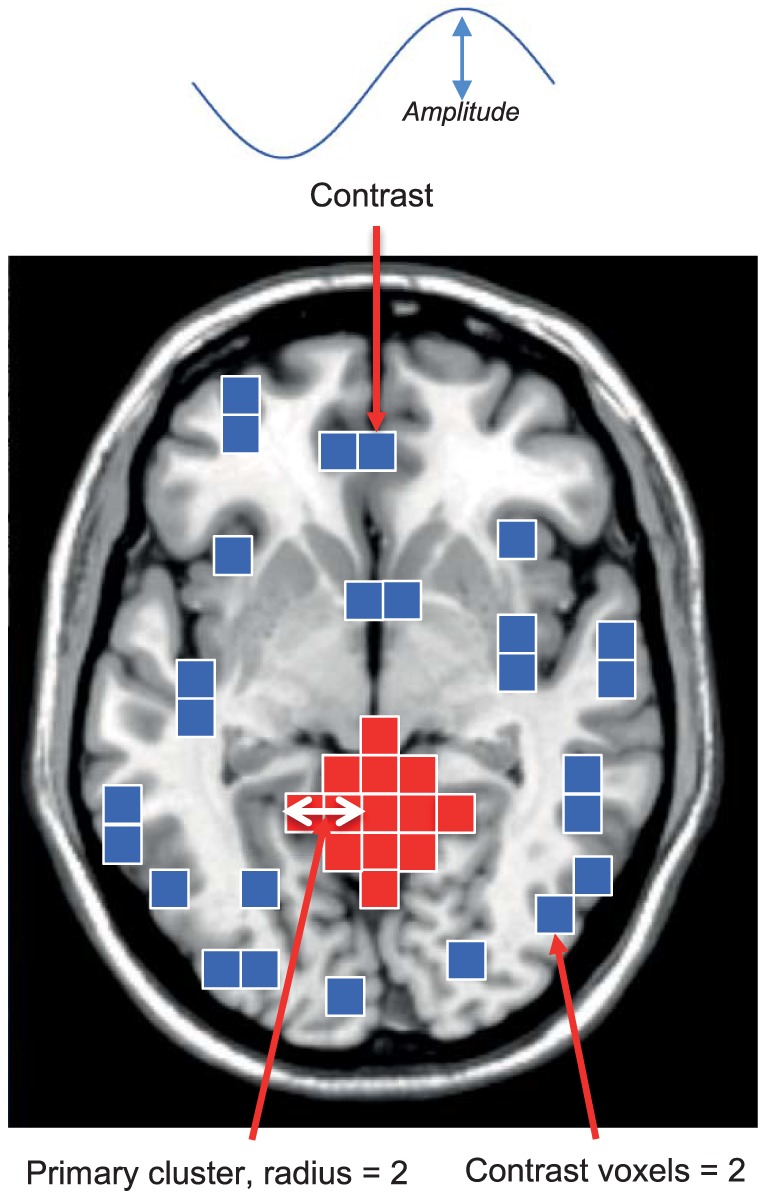
A figure displaying contrast properties which were altered during the simulation. ROC curves were calculated as the contrast parameters illustrated above were varied. The performance of methods was assessed as four parameters were varied: **t –** the initial threshold, **CN –** the contrast to noise, **R –** the radius of the primary cluster and **CV –** the number of contrast voxels outside of the primary cluster, as a multiple of the number of voxels in the primary cluster.

The process used to construct ROC curves is described in detail below:

1. Two groups filled with Gaussian random noise were constructed. Each group consisted of twelve ‘scans’. For each subject, timecourses of Gaussian random noise were generated for voxels in a single slice of the study grey matter mask; timecourses were equal in length to the experimental normocapnia/hypercapnia scans. These two groups are subsequently referred to as groups A and B.

2. A contrast + noise group was also created. Here, a time-varying sine curve was added to the timecourses of a subset of ‘contrast’ voxels in group A (see fig. 4). The sine-curve had a frequency of 0.1 Hz, this is around the same frequency as BOLD based signal change [35]. The contrast was added to a large spatially restricted cluster: this group of voxels is termed the ‘primary cluster’. The contrast was also added to voxels scattered throughout the rest of the slice (see fig. 1c, fig. 4). This group is subsequently referred to as group C.

3. A temporal autocorrelation structure matching that of the real data was added to voxel timecourses across subjects and groups. Data was then spatially smoothed using an 8×8 mm Gaussian kernel and low-pass filtered at a frequency of 0.1 Hz.

4. Both methods were used to calculate the true positive rate of primary cluster detection between groups A and B. TPR was calculated as |C ∩ ĥ|/|C| where C is the set of voxels associated with the primary cluster, and ĥ is the set of voxels which form clusters above the discrimination threshold. The true positive rate was calculated as a function of the discrimination threshold.

5. The FPR was calculated between groups B and C. FPR = 1 if |ĥ|≥1, FPR = 0 if | ĥ| = 0, where V denotes all voxels under analysis. The false positive rate was calculated as a function of the discrimination threshold.

6. Steps 1-5 were repeated a thousand times for each condition. The mean TPR and FPR were calculated across trials for each discrimination threshold. They were then plotted against one another.

The performance of the CSS and CMS methods was evaluated under different conditions by changing methodological parameters and contrast properties. The efficacy of CSS and CMS were tested as three contrast properties were varied: **CN –** the contrast to noise (CN is given by the formula: A/σ_N_ where A is the amplitude of the sine wave comprising the contrast, and σ_N_ is the standard deviation of the noise [36]), **R –** the radius of the primary cluster and **CV –** the number of contrast voxels outside of the primary cluster, as a multiple of the number of voxels in the primary cluster. The contrast properties that were varied are illustrated in fig. 4. CMS and CSS performance was also assessed at several initial threshold values. Performance was only evaluated for FPR values ranging from 0 to 0.05. FPR values higher than 0.05 are not generally of interest in neuroimaging studies.

### Simulation results

**Figure 5:**
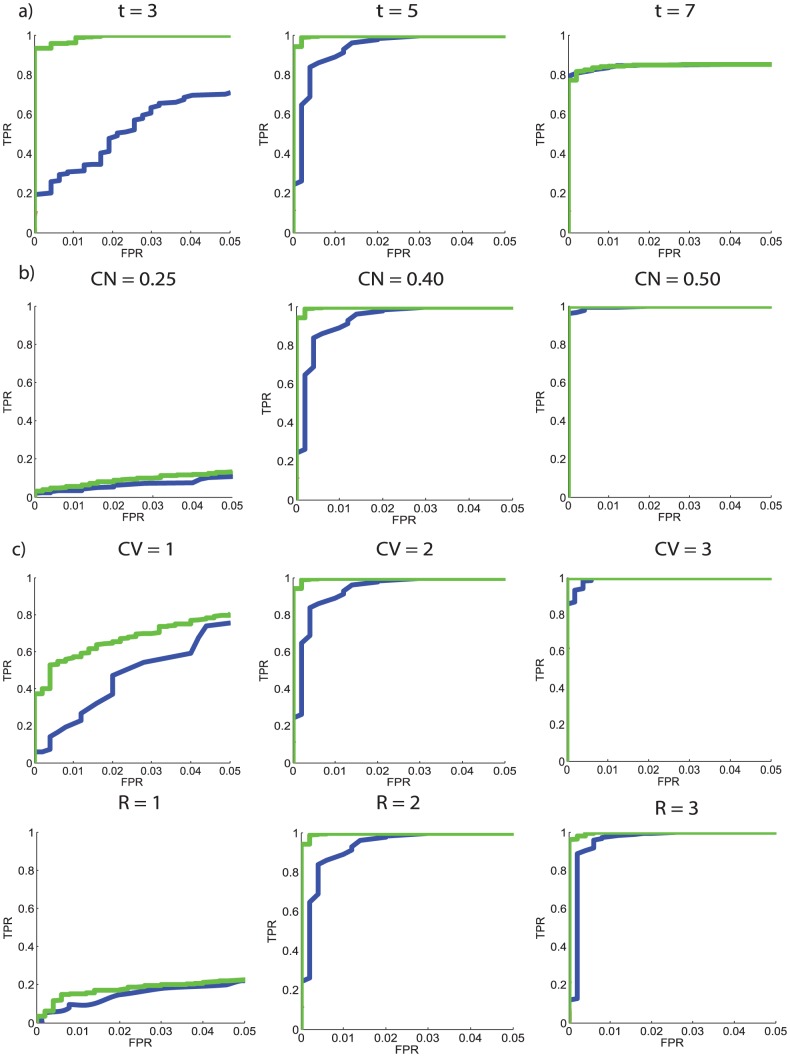
ROC curves for each of the comparison methods, plotted from simulations carried out under different experimental conditions. ROC curves illustrating the performance of CSS and CMS methods as contrast and methodological properties were varied. 5a) shows how varying the initial threshold can effect performance 5b)shows the effect of contrast to noise on method performance 5c) shows how varying the number of contrast voxels changes performance 5d) illustrates how altering the primary cluster radius can effect performance. Green curves: CMS, Blue curves: CSS.

Fig. 5a shows the performance of CSS/CMS methods as the initial cluster threshold was varied. Initial thresholds of t = 3, t = 5 and t = 7 were applied to CSS and CMS methods. R, CV and CN were held constant at 2, 2 and 0.4 respectively. Both CSS and CMS were most effective when an initial threshold of t = 5 was applied to the simulated data. It should be noted that this doesn't mean that a t-value of 5 is associated with the best CSS/CMS performance generally. This t-value is only optimal under the very specific circumstances considered here. It is also worth noting that the simulation was carried out using 2-D data; it is likely that the ideal threshold would be higher if 3-D data was used.

Fig. 5b shows the ability of the comparison methods to detect the contrast as CN was varied. Realistic [36] CN values of 0.25, 0.4 and 0.5 were utilised. Other parameters were held constant at R = 2, CV = 2 and t = 5. As expected, the performance of each of the comparison procedures improved as the contrast amplitude was increased.

Fig. 5c shows the efficacy of each method as the number of contrast voxels outside of the primary cluster was altered. CV values of 1, 2 and 3 were used. R, CN and t were held constant at 2, 2 and 5 respectively. The performance of both methods improved as the number of contrast voxels outside of the primary cluster was increased.

Fig. 5d shows the performance of each of the methods as the size of the primary cluster was altered. Primary cluster radii of R = 1, R = 2 and R = 3 were utilised. CN, t and CV were held constant at 0.4, 5 and 2 respectively. As expected, method performance improved as R was increased.

These results illustrate that the performance of CMS is generally higher than that of CSS across the range of methodological and contrast properties used.

## Real Data, Materials and Methods

### Ethics statement

The study was approved by the local ethics committee (College Ethics Review Board, University of Aberdeen), ethical approval was confirmed online. Written informed consent was obtained from all participants.

### MRI acquisition

Measurements were carried out on a Philips 3T Achieva scanner (Philips Healhcare, Best, The Netherlands) using a 32-channel phased-array receiver coil. Gradient-echo EPI was used for the functional connectivity MRI (fcMRI). Imaging parameters were TE = 30 ms, TR = 2 s, flip angle = 78°, matrix size = 96×96, field of view = 240×240 mm^2^, number of slices = 32, slice thickness = 3.5 mm, parallel imaging method = SENSE, acceleration factor = 2, number of dynamic scans = 540, number of dummy scans = 4. In addition, a high-resolution T_1_-weighted structural scan was obtained using fast 3D gradient echo imaging. Scan parameters were TR = 8.2 s, TE = 3.8 ms, flip angle = 8°, matrix size = 240×240×125, field of view = 240×240×160 mm^3^, voxel size = 1.0×1.0×1.0 mm^3^, total acquisition time = 5 min 35 s.

### Participants

Twelve healthy volunteers (seven male; mean age and SD = 24±4 years) with no medical history of migraine, anxiety or any illnesses of the heart, brain, circulatory or respiratory systems were recruited. Participants who had a family history of subarachnoid aneurysm, subarachnoid haemorrhage, intracranial aneurysm or arteriovenous malformation were excluded.

### Inducing hypercapnia

Transient hypercapnia was induced by breathing a specialised gas mixture consisting of 6% CO_2_, 21% O_2_ and 73% N_2_ (BOC Healthcare, Manchester, UK). Subjects were positioned supine on the patient table and wore an anaesthetic facemask (QuadraLite, Intersurgical, Wokingham, UK). A specially designed unidirectional breathing circuit (Intersurgical, Wokingham, UK; product code: 2013014) was used to deliver either room air or the 6% CO_2_ mixture (this circuit is illustrated in fig. S1). This circuit included a reservoir that was continuously replenished with gas throughout the breathing cycle. CO_2_ gas was supplied from a cylinder attached to the breathing circuit via a length of plastic tubing. When switching from hypercapnia to normocapnia, the breathing circuit was flushed with medical air (21% O2, 79% N2) to clear out any residual CO_2_. The plastic tubing was then detached and the subject breathed room air through the facemask and breathing circuit. Subjects were asked to try on the facemask and breathing circuit prior to the scan, none of the subjects reported any discomfort or difficulty breathing. Physiological parameters (heart rate and arterial oxygen saturation) were continuously monitored using a pulse oximeter (Model 7500FO, Nonin Medical, Inc., Plymouth, Minnesota, USA). Respiratory CO_2_ concentration was continuously monitored using a carbon dioxide analyser (Model CD-3A, AEI Technologies, Pittsburgh, PA, USA). The analogue output signal was digitised using a standard analogue to digital converter and stored on a hard drive. The end-tidal concentration was extracted from the recorded respiratory data using a routine written in Matlab (The Mathworks, Natick, MA, USA).

### Experimental paradigm

Each scan started with two minutes where the participant was instructed to lie still and ‘think of nothing in particular'. This was followed by six minutes where visual and motor tasks were carried out. This task section was followed by two minutes where the screen went blank and the subject was again asked to do ‘nothing in particular'. The six minute task section was then repeated. Task sections were carried out under normocapnia and hypercapnia, the ordering of these sections was randomised across subjects to avoid any effects introduced by subject habituation to the scan environment. Functional connectivity was compared between the two, six-minute task sections carried out under normocapnic and hypercapnic conditions. These simple visual/motor tasks were used to keep the participants' attention focused during the scan. Tasks requiring only low cognitive demand have been argued to provide a more stable baseline in functional connectivity analyses, compared to a pure resting state condition [14], [37].

### Data pre-processing

Standard fMRI pre-processing was carried out using the Statistical Parametric Mapping software package SPM8 (http://www.fil.ion.ucl.ac.uk/spm). Pre-processing steps included realignment, slice-time correction, combined segmentation and spatial normalisation, and spatial smoothing using a 8 mm FWHM Gaussian kernel. Voxels in each volume were resampled from 2×2×2 mm to 4×4×4 mm to reduce computational complexity.

In order to further reduce computational complexity, a study specific, binary grey matter mask was created and applied to the data. This was achieved by averaging the individual, probabilistic grey matter maps produced during segmentation, and applying a probability threshold of 0.8 for inclusion within the mask. The resulting binary mask consisted of 9,083 grey matter voxels. All further analysis of the data was restricted to the grey matter voxels defined by the binary mask. Voxel timecourses were low-pass filtered (cut-off frequency = 0.1 Hz) and baseline corrected using a 2nd order cosine basis set to remove low-frequency signal drifts. Realignment parameters were used as covariates of no interest in a voxel-wise linear regression on each timecourse and the resulting residual signal timecourses were used in all further analyses.

### Functional connectivity analysis

Connectivity matrices – also known as similarity matrices – were calculated using Pearson's correlation coefficient for each participant and condition (hypercapnia, normocapnia) from the preprocessed timecourse data.

### Statistics based on element-wise comparison

**Figure 6:**
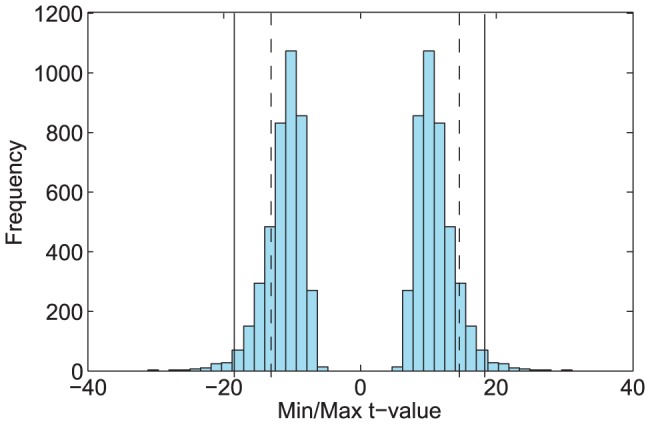
Distribution of maximum/minimum t-statistics across all permutations. Permutation distribution of maximum and minimum t-statistics across all matrix elements in a comparison between normocapnic and hypercapnic states. The solid lines represent p<0.05 FWE corrected significance thresholds. The dashed lines represent the maximum and minimum t-statistics in the experimental labelling.

Comparisons were made between normocapnic and hypercapnic connectivity matrices using a non-parametric paired t-test, for all 4096 permutations of the experimental labelling. As explained in the theoretical background section, the full permutation distribution can be calculated using half of all permutations of the experimental labelling. Here, all permutations were used to illustrate the symmetry of the labellings (see fig. 6).

Both maximum and minimum element-wise t-values were recorded for each permutation, to produce distributions of maximum/minimum t-statistics. Element-wise t-values in the actual, experimental labelling were then compared to these distributions. T-values in the top or bottom 2.5 percentiles of the distribution can be declared significant at the 0.05 FWE corrected level. Statistics in the top or bottom 2.5 percentile, rather than the top or bottom 5 are considered significant as the test carried out was two-sided.

### Statistics based on CSS and CMS

The procedure described in the theoretical background section was used to help guide the initial threshold choice. An initial threshold associated with a critical cluster size of nineteen was chosen based on the following consideration: Due to spatial smoothness in the data (voxel size 4×4×4 mm; smoothing kernel 8 mm), each voxel under analysis will share a substantial amount of information with its nearest neighbours. A critical cluster size of nineteen was chosen as this number includes a voxel and its nearest neighbours, connected by a face or an edge. Therefore, any significant CSS clusters in the experimental data will utilise the spatial correlation in the data, whilst maintaining a high degree of spatial specificity. It should be noted that it is possible for statistically significant CMS clusters in the experimental labelling to have an extent smaller than nineteen voxels. However, this is unlikely due to the spatial smoothness in the data.

Fig. 3 shows a plot of the initial t-threshold against the critical cluster size. The plot was fitted with a cubic spline curve. A cluster-forming t-threshold of 9.3 was interpolated from the curve as being associated with a critical cluster extent of nineteen voxels. The permutation distributions, which were used to calculate the critical cluster sizes in the plot, used a thousand permutations of the experimental labelling. The full permutation distribution was then calculated for CSS and CMS statistics at this threshold, using the procedure outlined in the theoretical background section. Clusters in the experimental labelling were then compared to this distribution to assess their FWE corrected significance.

## Experimental Results

Inhalation of 6% CO_2_ increased the across subject mean end-tidal-CO_2_ from 42.9±1.8 mm Hg to 52±1.7 mm Hg. These values were calculated by computing the mean Et-CO_2_ value across normocapnia/hypercapnia sections, then across subjects. All subjects tolerated this well; no side effects were reported by any of the participants.

Fig. 6 shows the distribution of maximum and minimum t-statistics calculated from the element-wise comparison of connectivity matrices. The largest t-value across all matrix elements when comparing between normocapnic and hypercapnic states was 14.35, the smallest was −12.27; these values are shown as dashed lines in fig. 6. Neither of these changes was significant at the p<0.05 FWE corrected level when compared to the distribution of maximum/minimum t-statistics; the maximum and minimum t-values corresponding to this level of significance are shown as solid lines. As can be seen from fig. 6, the distribution of the maximum t-statistic is symmetric with respect to positive and negative t-values.

**Figure 7:**
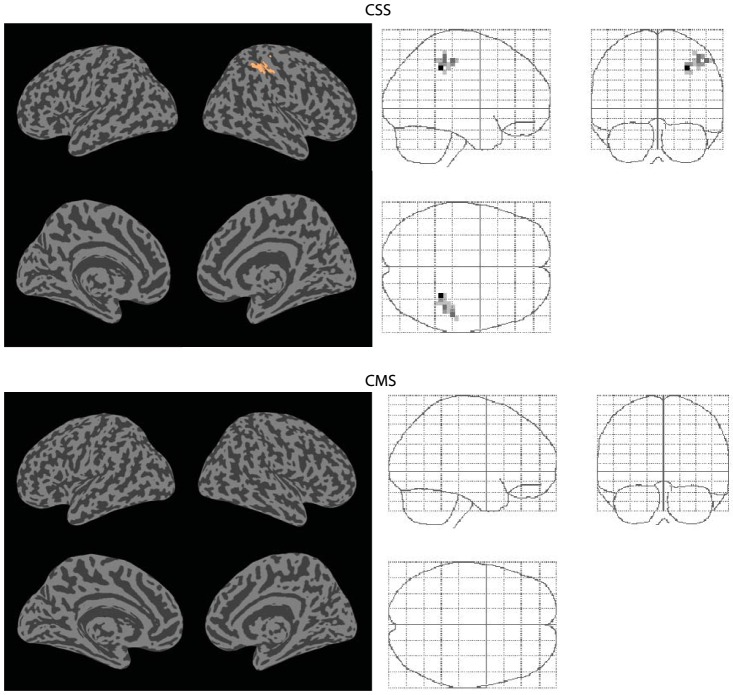
Connectivity decreases. Significant decreases (p<0.05 FWE corrected) in functional connectivity between normocapnia and hypercapnia, identified by the CSS and CMS statistics.

**Figure 8:**
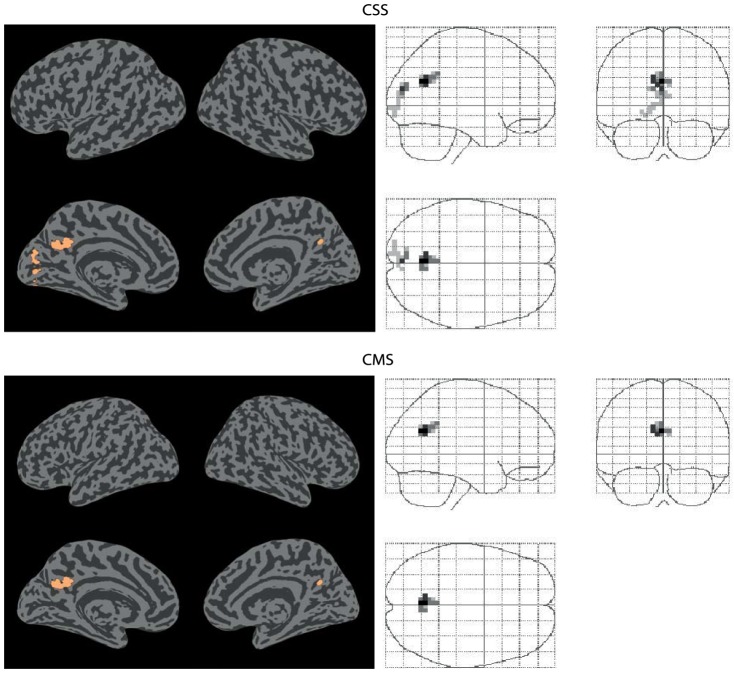
Connectivity increases. Significant (p<0.05 FWE corrected) increases in functional connectivity between normocapnia and hypercapnia identified by the CSS and CMS statistics.

In contrast to the element-wise comparison, the methods introduced in this paper (CSS, CMS) identified both significant increases and decreases (p<0.05, FWE corrected) in functional connectivity associated with the hypercapnic state. The results are shown in figs. 7 and 8; the spatial location and statistical significance of clusters are shown in tables 1 and 2. Both methods detected decreases in functional connectivity in the posterior cingulate cortex (BA 23). CSS detected an additional decrease in connectivity in the primary visual cortex (BA 17). CSS also detected an increase in connectivity in the supramarginal gyrus (BA 40). CMS detected no increases in connectivity.

**Table 1:** Decreases in connectivity.

Method	Cluster number	Statistic size	FWE corrected significance	Brodmann area	MNI co-ordinates
CSS	CSS1	26	0.039	23	−2 −58 27
	CSS2	24	0.041	17	−11 −92 −2
CMS	CMS1	46	0.042	23	−2 −58 27

MNI coordinates, statistic size and significance of all p<0.05 FWE corrected decreases in global connectivity between normocapnia and hypercapnia, identified by the CSS and CMS statistics.

**Table 2:** Increases in connectivity.

Method	Cluster number	Statistic size	FWE corrected significance	Brodmann area	MNI co-ordinates
CSS	CSS7	22	0.049	40	40 −35 47

MNI coordinates, statistic size and significance of all p<0.05 FWE corrected increases in global connectivity between normocapnia and hypercapnia, identified by the CSS and CMS statistics.

**Figure 9:**
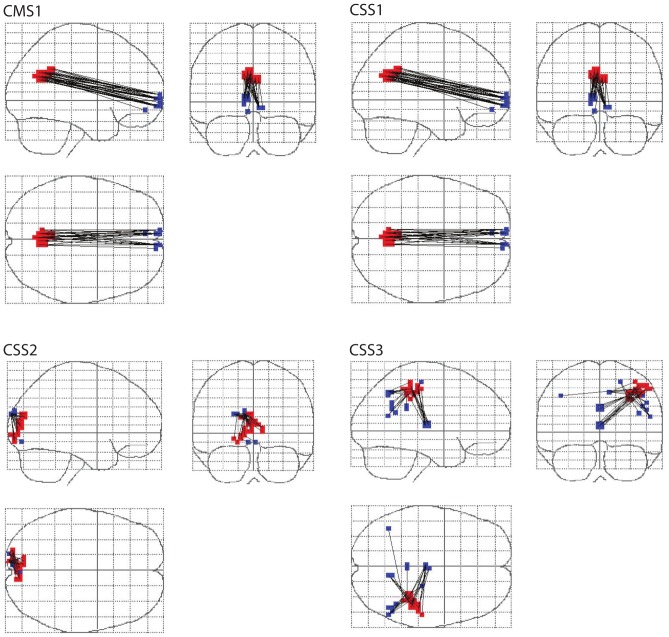
Networks of connectivity change. Altered functional connectivity identified by the CSS and CMS statistics (red) with the pseudo thresholded changes that contribute to these clusters (blue). It should be noted here that only whole clusters, rather than individual connections, can be deemed to be significant.

Each of the clusters of altered connectivity identified by CMS and CSS is associated with pseudo thresholded changes across the cortex. These changes can be displayed in image space. Fig. 9 shows the primary clusters (red) and the connected voxels which contribute to these clusters (blue). In analogy to other cluster-based methods, the FWE-corrected significance level applies to the network component as a whole (i.e., red and blue voxels in fig. 9) rather than to individual connections between pairs of voxels.

Running both of the whole brain comparison tests introduced in this paper (CSS, CMS) required less than 22 hours of computation time on an Linux machine with 48 GB of memory and eight cores, each with 2.56 GHz of processing power. Most of this time was spent in calculating the matrix of t-statistics for each permutation of the experimental labels. Once this matrix was determined, calculating each of the cluster based statistics from the matrix required a negligible amount of computation time. For this reason, running one of the two tests separately would have required approximately the same amount of time as running them both together.

## Discussion

### CSS and CMS methods

Both CSS and CMS detected changes in global connectivity between experimental conditions, while the element-wise comparison of connectivity matrices identified no significant changes when corrected for multiple comparisons (p<0.05 FWE corrected). Similar to conventional clustering methods in fMRI, CSS and CMS make use of the spatial smoothness in the data to provide an increase in statistical power. Spatial smoothness arises from intrinsic factors such as the spatial extent of the BOLD signal [38] and to a lesser degree, spectral leakage of the Fourier transform during image reconstruction. Additional spatial smoothing is often applied to fMRI data by means of spatial filtering in order to improve the contrast-to-noise ratio and to reduce the effects of imperfect inter-subject coregistration.

Overall, CSS detected three clusters of significant connectivity change, whilst CMS detected one. Significance in CSS is determined by spatial extent, this method is therefore sensitive to spatially extended clusters. CSS detected two clusters of altered connectivity that CMS was unable to recognize. These clusters were large by volume but of low intensity, leading to lower CMS significance values relative to CSS. CMS has the potential to detect clusters that are smaller by volume but more focal in intensity than the clusters identified by CSS. As can be seen from the simulation results, CMS is generally more sensitive than CSS. However, in the present investigation, CMS did not detect any changes that CSS was unable to identify. Note that in this context, ‘intensity’ refers to the number of connectivity changes between a voxel, and all other voxels in the brain (see Theoretical Background).

CSS and CMS are capable of identifying clusters of significantly altered global connectivity. These clusters are associated with pseudo thresholded connectivity changes across the brain (cluster-links). Changes between a primary cluster and the rest of the brain may be widely distributed across the cortex, or localised to just a few areas. These different patterns of connectivity change can be seen in fig. 9. The primary cluster in CSS1 (red) mainly shows changes with one other area of the brain (blue). In contrast, the primary cluster in CSS3 (red) shows changes that are widely distributed across the brain (blue). Taken individually, these changes cannot be considered significant: only weak control of the family-wise error rate is maintained at the single connection level. Only the cluster based network taken in its entirety can be declared significantly altered between conditions.

In seed based connectivity mapping, connectivity is calculated between a single cortical region, and the rest of the brain. When connectivity maps are compared between groups or conditions, it is possible to identify cortical regions where connectivity is significantly altered with the seed used, with strong control over connection-wise changes. CSS and CMS methods are closely related to analyses involving the comparison of seed based connectivity maps. In CSS and CMS, connectivity matrices are compared between groups or conditions. Each column of the connectivity matrix is equivalent to a seed based map, associated with a distinct voxel ‘seed’. This initial comparison can be thought of as a test between all possible connectivity maps in the brain. CSS and CMS are defined from connectivity changes exceeding the initial threshold in spatially contiguous voxels. Fig. 9 shows patterns of connectivity change between a spatially distinct cluster, and the rest of the cortex. As has been discussed, CSS and CMS only provide weak control over the family-wise error rate at the single connection level; when seed based connectivity maps are compared, strong control over connection-wise changes is maintained. CSS and CMS can be used as exploratory, data driven methods to guide the hypotheses of future studies: Primary clusters showing a significant change in connectivity can be used as seeds in future analyses. A follow up seed based analysis has not been carried out on the present data, as this would constitute ‘double dipping’ [31](Using the same data for both selection and selective analysis) and is not statistically valid. To avoid this issue, an independent dataset taken under identical conditions is required to provide connection-wise control over the family-wise error rate.

The Network based statistic (NBS) is sensitive to connectivity changes that form altered network components. The main problem with NBS is that connectivity changes cannot be localised to a single area of the brain. Furthermore, NBS does not model the intrinsic spatial smoothness of BOLD fMRI data, which makes it less suitable for applications on a voxel-by-voxel level. The methods introduced here are less complex and require less computational time than spatial pairwise clustering (SPC). SPC demands the initialisation of a very large (N^2^-N)/2×(N^2^-N)/2 matrix [39], which in the present investigation would contain approximately 10^17^ elements. SPC is sensitive to large, pairwise clusters of altered connectivity. As noted by Zalesky et al (2012), smaller pairwise clusters may not be identified using this method [24]. It is important to note that although there are general advantages and disadvantages associated with all of the connectivity clustering procedures discussed, these methods can be regarded as complementary, as they measure completely different kinds of connectivity change. This is similar to the way in which different graph theoretical measures (e.g. centrality, path length) quantify completely different aspects of network topology.

A challenge CSS and CMS share with other cluster-based methods such as SPC and NBS, is the need to specify a cluster-forming threshold. While this is not a problem from a statistical point of view, the initial threshold choice will affect the sensitivity and spatial specificity of the method. In this investigation, a procedure was outlined that helps guide the initial threshold choice. However, it is important to note that this procedure does not circumvent all the problems associated with the initial threshold choice. The somewhat arbitrary nature of this choice remains an issue, as it does for all cluster-based methods.

Meskaldji et al have proposed an alternative framework for the comparison of connectivity matrices; this procedure is termed subnetwork based analysis (SNBA) [40]. In this method, the whole brain network is divided into subnetworks, a meaningful summary statistic is then calculated for each subnetwork. Type 1 error control is carried out at the subnetwork level. It would be interesting to see how the sensitivity of SNBA compares to that of CSS/CMS.

In this investigation, maximum CSS and CMS statistics were calculated for positive and negative cluster forming thresholds, at each of the 4096 possible experimental permutations. Similarly, maximum and minimum t-values were calculated across all connectivity values for each permutation of the experimental labels. As noted in the theoretical background section, it is possible to produce the full permutation distribution for each statistic from half of all possible relabellings. Constructing the full permutation distribution in this way would almost half the computation time required by all methods.

CSS and CMS were applied to compare functional connectivity between different experimental conditions (i.e., hypercapnia and normocapnia). Both methods can easily be adapted to allow for a comparison between multiple experimental conditions or between different groups of subjects by replacing the paired t-statistic (see Methods) with any other suitable statistic (e.g., two-sample t-statistic for a comparison of two different groups of subjects). In the implementation presented here, a non-parametric permutation test was used for statistical significance testing. An advantage of this approach is that is does not make any assumptions about the nature of the underlying null distribution.

The connectivity analysis described here was based on BOLD fMRI data. However, CSS and CMS methods could equally well be applied to other modalities which are capable of producing connectivity data, for example: electroencephalography or diffusion tensor imaging.

### Hypercapnia

The primary objective of using hypercapnia in this study was to induce transient changes in functional connectivity in order to test the sensitivity of the CSS and CMS methods, rather than to investigate any specific effects of hypercapnia on functional connectivity or to study the mechanisms underlying BOLD fMRI. However, the findings presented here may have implications for the experimental design of calibrated fMRI studies, and are therefore discussed in more detail in this section.

The BOLD effect relies on the variation of magnetic susceptibility in brain tissue caused by changes in blood oxygenation. Because the BOLD effect is not a direct measure of neuronal activity, the signal measured is subject to non-neuronal, physiological confounds. Calibration methods utilising the hypercapnic state are theoretically able to remove many of these confounding effects [41]–[43]. However, calibrating the BOLD signal in this way relies on the assumption that CO_2_ itself does not effect neuronal activity. The accuracy of this presumption is an area of long-standing contention, with the first study being published in 1948 and the issue still unresolved [44], [45].

The neural response to the hypercapnic challenge is difficult to characterise using BOLD fMRI. CO_2_ is a potent vasodilator and therefore has an effect on the BOLD signal that is at least partly independent of any metabolic demands [46]. Global cerebral blood flow and BOLD signal are increased during hypercapnia. This means that, taken alone, changes in BOLD signal observed during hypercapnia do not necessarily suggest a change in neuronal activity. Despite the fact that CO_2_ can cause changes in the BOLD signal, which are independent of any change in neural function; some of the results obtained in the present investigation are difficult to account for without invoking a neuronal mechanism.

In this study, both increases and decreases in functional connectivity were observed during hypercapnia (see Table 1). In a connectivity analysis restricted to the primary motor areas, Biswal et al. (1997) observed a reduction in functional connectivity between left and right motor cortices under conditions of moderate hypercapnia (induced through the inhalation of 5% CO_2_ gas) [26]. Similarly, Xu et al [25] found that functional connectivity was reduced between the posterior cingulate cortex and other default mode network areas during moderate hypercapnia. This is consistent with the results obtained in the present investigation. Clusters CSS1 and CMS1 are located in the posterior cingulate cortex and showed a significant decrease in connectivity with other default mode areas. This study also found functional connectivity decreases in other brain areas (i.e., clusters CSS2 and CSS3, see Table 1), which were not reported by Xu et al. (2010). This is explained by the fact that the functional connectivity analysis undertaken by Xu et al. (2010) was restricted to the default mode network, whereas the analysis in this study encompassed the whole brain.

Generally, a decrease in functional connectivity under hypercapnic conditions can be explained by a decrease in the relative vascular response to metabolic demands as originally proposed by Biswal et al. (1997). However, this mechanism cannot explain the observed increases in functional connectivity during hypercapnia (exhibited by cluster CSS3), unless the increase in functional connectivity is driven by a reduction in anticorrelation. Further analysis provided no evidence for decreases in anticorrelation, and showed that the observed changes were genuine, absolute increases in connectivity.

While the observed increases in functional connectivity may suggest changes in neuronal activity, this hypothesis cannot be proven on the basis of current data as this inference is indirect. In this study, the hypercapnic state was induced through the inhalation of a fixed concentration of CO_2_ gas. Wise et al have shown that this method of inducing hypercapnia is associated with a change in arterial O_2_; this leads to fluctuations in the BOLD signal that are not accounted for [47]. Although this confound is unlikely to have a significant impact on this group analysis, the indirect nature of the modality makes interpretation difficult.

To help account for these problems, an interesting prospective area of study would be a simultaneous EEG-fMRI resting state investigation, taken under normal and hypercapnic conditions. A study of this type would be able to take advantage of the superior spatial resolution of fMRI whilst utilising EEG as a more direct measure of neuronal activity. As the level of neuronal activity is closely linked to the cerebral metabolic rate of oxygen (CMRO_2_), a confirmation of altered neuronal activity during hypercapnia would have direct implications for the experimental design of calibrated fMRI studies [41]-[43], which rely on the assumption that CMRO_2_ is unaffected during moderate hypercapnia.

## Supporting Information

Figure S1 **Schematic diagram of the breathing circuit.**
(PDF)
