# Correction: Cluster Size Statistic and Cluster Mass Statistic: Two Novel Methods for Identifying Changes in Functional Connectivity Between Groups or Conditions

**DOI:** 10.1371/journal.pone.0103658

**Published:** 2014-07-21

**Authors:** The PLOS ONE Staff

The images for Figure 7 and Figure 8 were inadvertently swapped. Please view the correct images and legends for Figure 7 and Figure 8.

**Figure 7:**
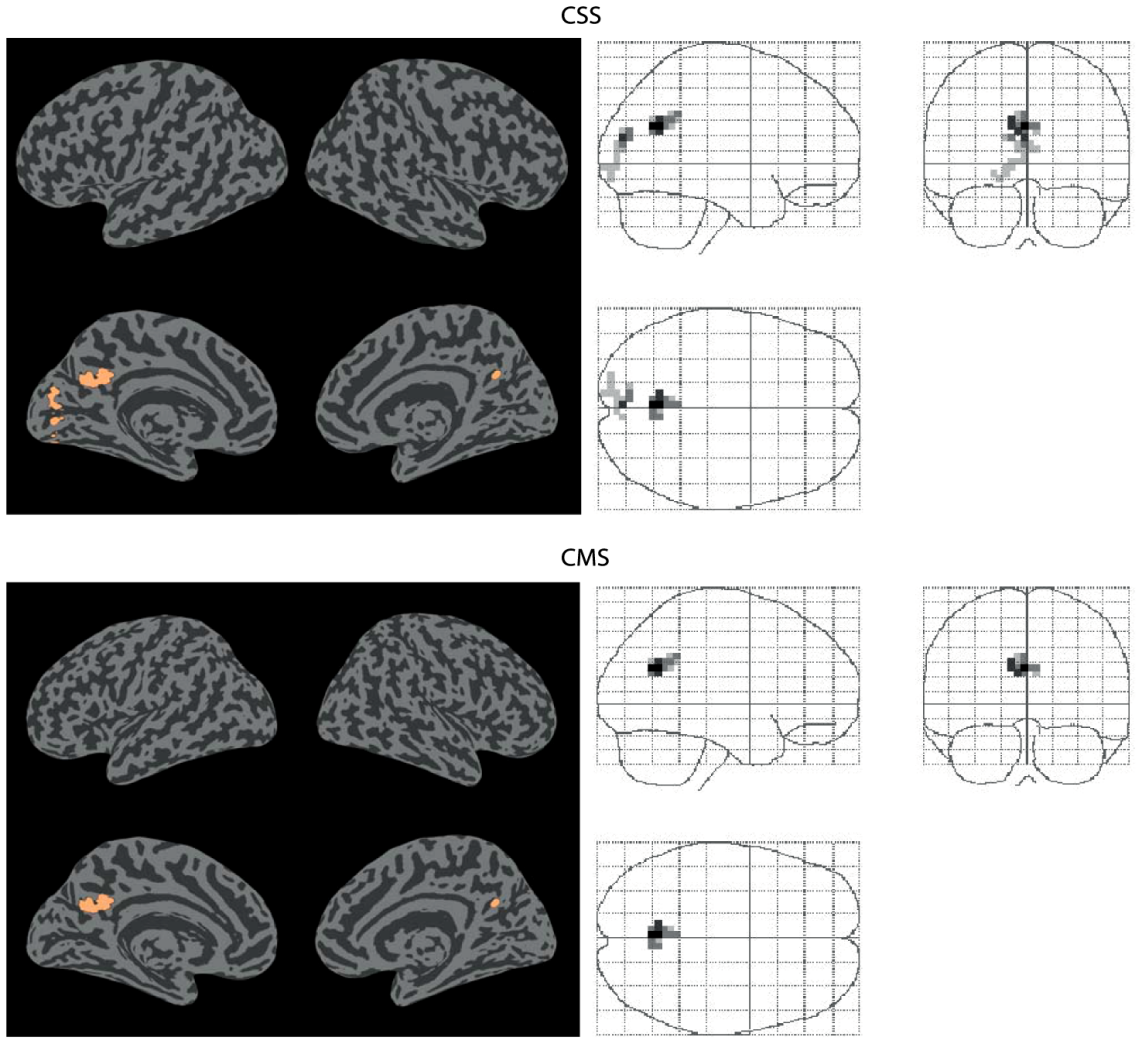
Connectivity decreases. Significant decreases (p<0.05 FWE corrected) in functional connectivity between normocapnia and hypercapnia, identified by the CSS and CMS statistics.

**Figure 8:**
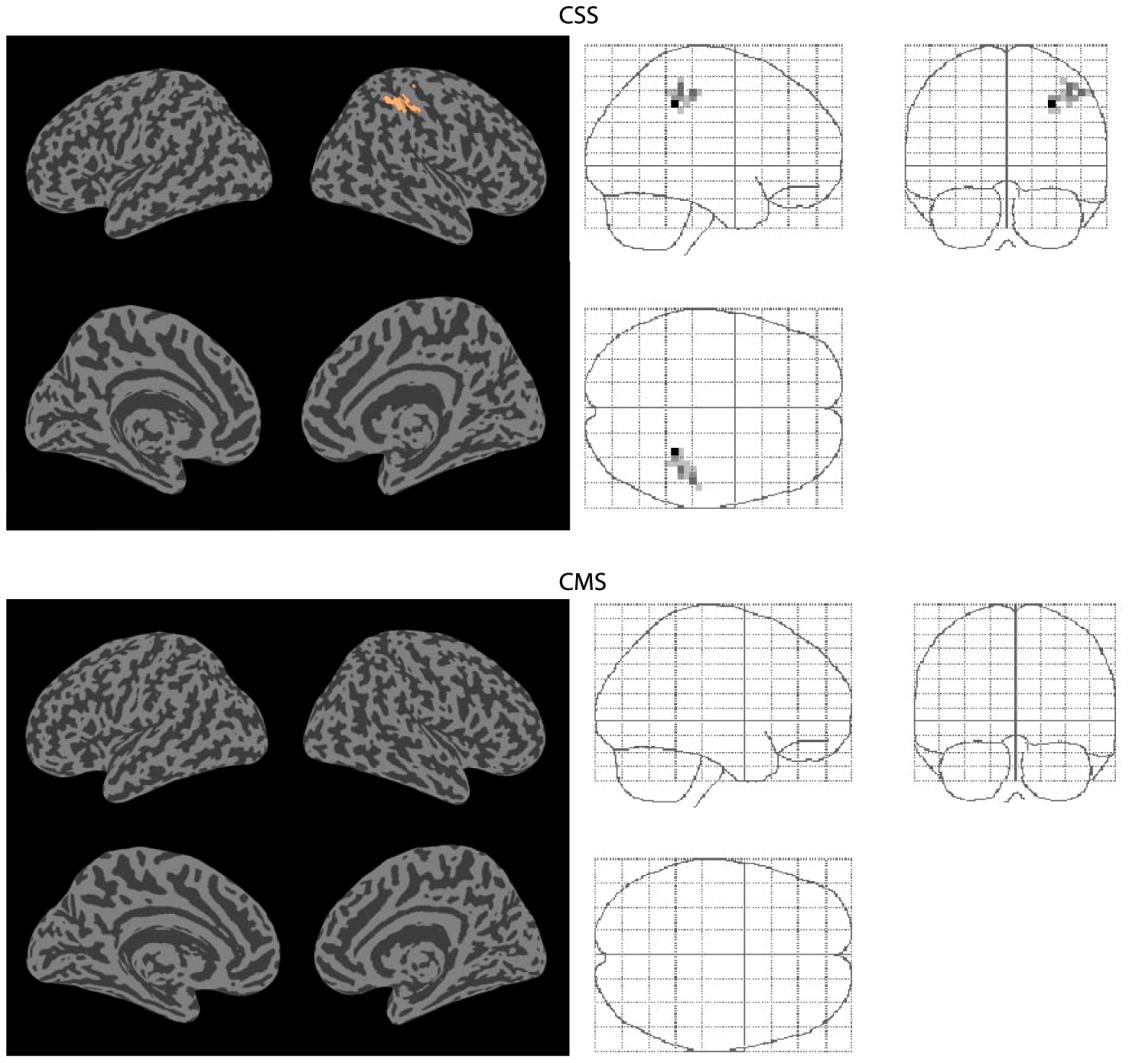
Connectivity increases. Significant (p<0.05 FWE corrected) increases in functional connectivity between normocapnia and hypercapnia identified by the CSS and CMS statistics.
