# Supplementary figures and images for: Cluster Size Statistic and Cluster Mass Statistic: Two Novel Methods for Identifying Changes in Functional Connectivity Between Groups or Conditions
Source: PLoS One. 2014 Jun 6;9(6):e98697. doi: 10.1371/journal.pone.0098697 (PMC4048154; doi:10.1371/journal.pone.0098697)

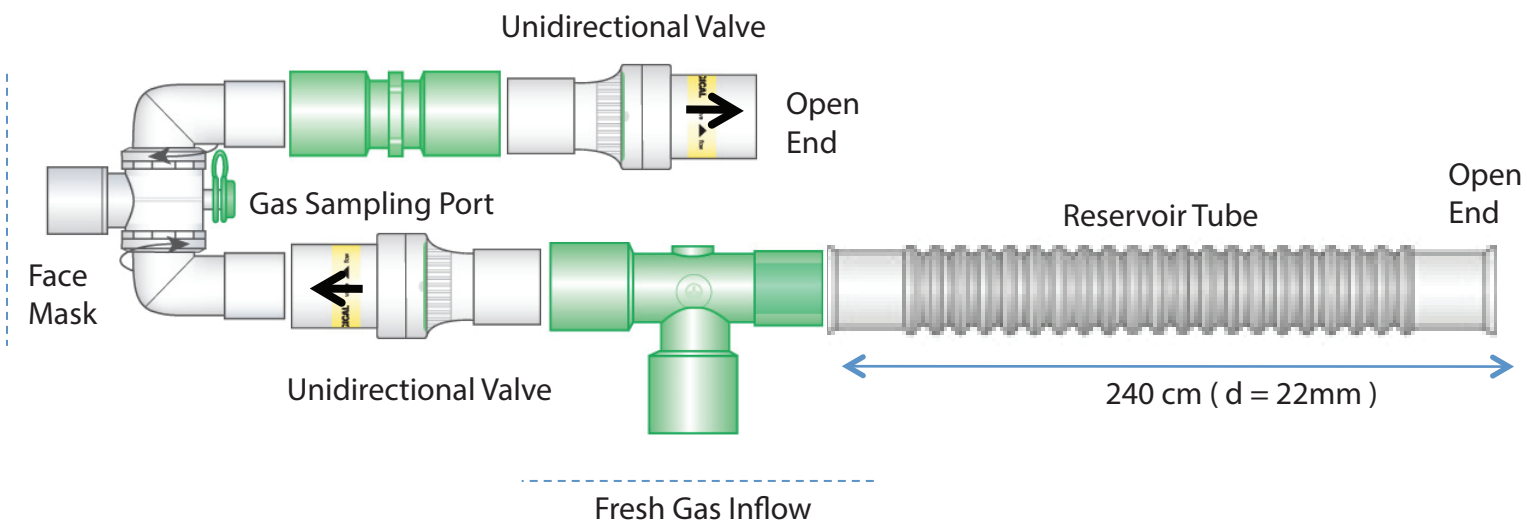

Supplement: Figure S1 — Schematic diagram of the breathing circuit. (PDF) [file pone.0098697.s001.pdf]
